# Nuclear Magnetic Resonance-Based Metabolomic Analysis Reveals Physiological Stage, Breed, and Diet Effects on the Intramuscular Metabolism of Amino Acids and Related Nutrients in Pigs

**DOI:** 10.3389/fvets.2021.681192

**Published:** 2021-08-10

**Authors:** Yingying Liu, Qinghua He, Md. Abul Kalam Azad, Yi Xiao, Yulong Yin, Xiangfeng Kong

**Affiliations:** ^1^Key Laboratory of Agro-Ecological Processes in Subtropical Region, Hunan Provincial Key Laboratory of Animal Nutritional Physiology and Metabolic Process, National Engineering Laboratory for Pollution Control and Waste Utilization in Livestock and Poultry Production, Institute of Subtropical Agriculture, Chinese Academy of Sciences, Changsha, China; ^2^Hunan Provincial Key Laboratory of Conservation and Genetic Analysis of Local Pig Breeds Germplasm Resources, Hunan Institute of Animal and Veterinary Science, Changsha, China; ^3^Department of Food Science and Engineering, College of Chemistry and Environmental Engineering, Shenzhen University, Shenzhen, China; ^4^College of Information and Intelligence, Hunan Agricultural University, Changsha, China

**Keywords:** amino acid, diet, fatty acids, metabolomic, physiological stage, pig

## Abstract

Skeletal muscle is a complex tissue that exhibits considerable plasticity in response to nutrients, animal, or its growth stage, but the underlying mechanisms are largely unknown. This study was conducted to evaluate the effects of physiological stage, breed, and diet on the metabolome of the skeletal muscle of pigs. Ninety-six barrows, including 48 purebred Bama mini-pigs, representing the fat type, and 48 Landrace pigs, representing the lean type, were randomly assigned to either a low- or adequate-protein diet (*n* = 24 per group). The experimental period commenced at 5 weeks of age and extended to the finishing period. *Psoas major* muscles (PMMs) were collected at the nursery, growing, and finishing stages; and the contents of amino acids (AAs), fatty acids (FAs), and metabolites were analyzed using a nuclear magnetic resonance-based approach. Results showed that most AAs and monounsaturated FAs (MUFAs; including C16:1 and C18:1) contents were increased (*p* < 0.05) gradually, while those of polyunsaturated FAs (including C18:2, C20:4*n*−6, C20:5*n*−3, and C22:6*n*−3) were decreased (*p* < 0.05) in the PMM with increasing age. Compared with Landrace pigs, Bama mini-pigs had higher (*p* < 0.05) contents of flavor-related AAs (including methionine, phenylalanine, tyrosine, leucine, and serine) in the nursery and growing stages and higher (*p* < 0.05) percentages of saturated FAs and MUFAs throughout the trial. Dietary protein levels affected the muscular profiles of AAs and FAs in an age-dependent manner. In addition, the adequate-protein diet increased (*p* < 0.05) the muscular contents of α-ketoglutarate in the two breeds. These findings indicate that the dynamic profiles of AAs, FAs, and metabolites in pig muscle tissues are regulated by breed, diet, and physiological stage.

## Introduction

Meat is an important part of the human diet, the main component of which is muscle tissue. Skeletal muscle is a complex tissue that exhibits considerable plasticity in response to the dietary intake of protein and energy. The characteristics of muscle regulate the quality and processing properties of meat. Body composition and its steady balance play an important role in animal health, especially the composition and metabolites of muscle. In the process of metabolism, organisms produce different metabolites, which in turn affect the metabolism of the body. Several metabolites, such as lactic acid, ethanol, and antimicrobial peptides, are the intermediate metabolites of microbes under special conditions. The final products of protein metabolism are water, carbon dioxide, and nitrogen-containing substances (e.g., urea). Regulating the rules and conditions of metabolism is beneficial to the production of these metabolites.

Metabolomics is a valuable tool to investigate the changes in metabolic regulations and then link these changes to the phenotypic outcome (1). Metabolomics can bring insights into metabolic fingerprints of any biological sample, quantify and measure the metabolic responses to the pathophysiological stimuli or genetic modifications, and lead to an integrated comprehension of metabolites function in health and disease (2). In addition, metabolomics provides a useful system for understanding global changes of metabolites in response to alterations in animals' genetics, nutrition, environmental conditions, and gut microbiota (3–5). The quantity and dynamic changes in the small molecular metabolites of cells, tissues, or organs can reflect the end point of the physiological regulation process in real-time. Therefore, metabolomics studies the “end point” of biological responses. The information obtained from metabolome analysis is closely related to the biological phenotype or the complete state of the organism. Therefore, the metabolome is the final expression of all biological phenomena (6).

Our previous studies determined the effects of dietary nutrient levels on growth performance and meat quality in two breeds of pigs, which found that meat quality was associated with both the breed and physiological stages of pigs (7). Feeding strategies can change several aspects of meat quality, other than eating quality, by affecting the muscle/fat ratio and meat composition. However, it is unclear how dietary nutrients intake and the physiological stage of the animal affect the profiles of amino acids (AAs) and fatty acids (FAs) in the muscle, as well as the metabolome, in different pig breeds. Thus, the present study aimed to evaluate the effects of dietary nutrient levels, breeds, and physiological stages on the contents of AAs and FAs and the metabolome of pig's skeletal muscle using a nuclear magnetic resonance (NMR)-based approach, also providing information on physiological regulation mechanisms.

## Materials and Methods

### Animals and Treatments

Ninety-six barrows, including 48 purebred Bama mini-pigs, representing the fat type [average initial body weight (BW) = 3.38 ± 0.96 kg], and 48 Landrace pigs, representing the lean type (average initial BW = 7.68 ± 0.89 kg), were fed from 5 weeks of age to the finishing stage. The experiment was conducted using two breeds (Bama mini-pigs vs. Landrace pigs) and two dietary nutrient levels [National Research Council diet (NRC diet) vs. Chinese conventional diet (GB diet)], thereby using a 2 × 2 factorial arrangement for the four treatment groups (Table 1). Each treatment group consisted of 24 randomly assigned piglets. The NRC diets were formulated to meet the NRC (2012) (8) recommended nutrient requirements, whereas the GB diets were formulated to meet the recommendations of the Chinese Feeding Standard for Swine (GB, 2004) (9). The ingredients and nutrient levels of each experimental diet are shown in Table 2. All animals were individually housed in 0.6 × 1.2 m pens with hard plastic slatted flooring. Each pen was equipped with a stainless-steel feeder and a nipple drinker. Room temperature was maintained at 25–27°C. All pigs had *ad libitum* access to drinking water and experimental diet and were fed three times daily (08:00, 13:00, and 18:00). The dietary stage was based on the physiological stage of pigs.

**Table 1:** Experimental design and treatments.

**Items**	**Landrace pigs**			**Bama mini-pigs**		
	**BW (kg)**	**GB diets**	**NRC diets**	**BW (kg)**	**GB diets**	**NRC diets**
Nursery stage	7–20	GB diet 1	NRC diet 1	3–15	GB diet 1	NRC diet 1
Growing stage	21–50	GB diet 2	NRC diet 2	16–35	GB diet 2	NRC diet 2
Finishing stage	51–90	GB diet 3	NRC diet 3	36–55	GB diet 3	NRC diet 3

*BW, body weight; GB diet, diet of Chinese Feeding Standard for Swine (2004); NRC diet, diet recommended by the National Research Council (2012)*.

**Table 2:** Ingredients and nutrient levels of experimental diets (%).

**Items**	**NRC diet 1**	**NRC diet 2**	**NRC diet 3**	**GB diet 1**	**GB diet 2**	**GB diet 3**
**Ingredients**						
Corn	62.80	66.00	69.50	63.00	60.00	66.00
Soybean meal, 42% CP	26.00	28.00	23.00	25.00	26.50	21.00
Fish meal, 62% CP	7.00	2.00	–	3.00	–	–
Wheat bran	–	–	3.00	6.34	10.75	10.50
Soybean oil	1.95	1.50	2.10	–	–	–
CaHPO_4_	0.45	0.70	0.65	0.80	0.80	0.50
CaCO_3_	0.50	0.50	0.45	0.56	0.65	0.70
Salt	0.30	0.30	0.30	0.30	0.30	0.30
Premix^a^	1.00	1.00	1.00	1.00	1.00	1.00
**Nutrient levels**						
Digestible energy (MJ/kg)	14.22	14.21	14.22	13.46	13.40	13.40
Crude protein^b^	20.06	18.01	15.11	18.03	16.05	13.46
Calcium	0.75	0.62	0.50	0.69	0.62	0.56
Available phosphorus	0.39	0.28	0.21	0.21	0.13	0.12

a *Premix provided for 1 kg of the complete diet: Cu (as copper sulfate), 10 mg; Fe (as ferrous sulfate), 100 mg; Se (as sodium selenite), 0.30 mg; Zn (as zinc oxide), 100 mg; Mn (as manganese sulfate), 10 mg; vitamin D_3_, 9.65 μg; vitamin A, 925.8 μg; vitamin E, 15.4 mg; vitamin K_3_, 2.3 mg; vitamin B_2_, 3.9 mg; calcium d-pantothenate, 15.4 mg; nicotinic acid, 23 mg; choline, 80 mg; vitamin B_12_, 0.016 mg*.

b *Crude protein was determined value, and other nutrients were calculated values*.

### Sample Collection

The BW ranges for the nursery, growing, and finishing stages were defined as 7–20, 21–50, and 51–90 kg, respectively, for Landrace pigs; and 3–15, 16–35, and 36–55 kg, respectively, for Bama mini-pigs (Table 1). At the end of each stage, eight pigs from each treatment group were randomly selected and weighted after 12 h of fasting. The pigs were then anesthetized and sacrificed by a jugular vein injection of 4% sodium pentobarbital solution (40 mg/kg BW) (10). After the head, legs, tail, and viscera were removed, the carcass was split longitudinally. Samples of the *psoas major* muscle (PMM) on the right-side carcass were immediately collected, and the visible intermuscular adipose tissue was carefully removed. Samples were snap-frozen in liquid nitrogen and stored at −80°C for further analysis.

### Determination of Amino Acids

To measure the contents of AAs in PMM, ~0.1000 g freeze-dried muscle was ground and hydrolyzed in 10 ml of hydrochloric acid solution (6 M) at 110°C for 24 h. The solution was then diluted with distilled water to 100 ml, and 1 ml of the supernatant was separated and used for analysis (11) in an ion-exchange AA analyzer (L8800, Hitachi, Tokyo, Japan) after filtering the samples through a 0.45-μm membrane (12).

### Determination of Fatty Acids

The percentages of FAs in intramuscular fat were determined by gas chromatography (GC) (10) in an Agilent 7890A system (Agilent Technologies, Santa Clara, CA, USA). Briefly, lipids were extracted from the PMM tissue using chloroform, and methyl esters were obtained *via* saponification with a solution containing 2 ml of hexane, 40 μl of methyl acetate, and 100 μl of sodium methoxide. After vortexing, the hexane layer was removed from the solution *via* anhydrous sodium sulfate, and FAs were determined by GC (chromatographic column sp-2560; 100 m × 250 × 0.2 μm) using the following conditions: initial column temperature, 140°C for 15 min; 3°C/min to 240°C; and 15 min at 240°C. The temperature of the injector and detector was at 250°C and the hydrogen flow rate at 30 ml/min, air at 400 ml/min, nitrogen at 40 ml/min, and carrier gas at 0.8 ml/min. The inlet temperature was 220°C, split ratio was 10:1, and injection volume was 1 μl. Individual FA peaks were identified by comparing their retention times with those of corresponding standards (Sigma Chemicals, St. Louis, MO, USA). Data are expressed as g/100 g of total identified FAs.

### Sample Preparation and NMR Spectroscopy

Approximately 50.00 mg of muscle tissues were separately extracted with 600 μl of cooled acetonitrile/water (1:1) using a biological sample homogenizer (Precellys 24; Bertin, Fontaine, France) at room temperature at 20 Hz for 90 s. Homogenates were sonicated for five cycles consisting of 1-min sonication and 1-min break in an ice bath. After centrifugation (12,000 rpm, 4°C) for 10 min, 500 μl of the supernatant was transferred to a 2-ml Eppendorf tube, while the insoluble residues were further extracted twice using the same procedure. The supernatants from the three extracts were combined, the acetonitrile was removed under vacuum, and then they were lyophilized. The obtained powder were reconstituted in 600 μl of phosphate buffer for NMR analysis (pH 7.4, K_2_HPO_4_/NaH_2_PO_4_ 0.15 M, 100% D_2_O).

The ^1^H NMR spectra of muscular extracts were acquired at 298 K using a Bruker Avance III 600-MHz NMR spectrometer (Bruker Corp., Billerica, MA, USA; operating at 600.15 MHz for ^1^H), equipped with an inverse cryogenic probe using a standard solvent-suppressed one-dimensional pulse sequence (recycle delay (RD)-90°-3 ms-90°-tm-90°-acquisition). These spectra were acquired and processed according to previously published parameters (3, 13). Briefly, the 90° pulse length (~10.0 μs) was adjusted individually for each sample. The 128 transients were collected into 32 k data points, with a spectral width of 20 ppm and a 2.0-s RD. A 50-Hz field irradiation was used to suppress the water peak.

### Analysis of NMR Data

Free induction decays were multiplied by an exponential window function of 1.0 Hz prior to Fourier transformation and corrected for phase and baseline distortions using TopSpin 3.2 (Bruker Corp.). A chemical shift was referenced to the peak of the doublet of α-glucose at δ 5.23.

The ^1^H NMR spectra (δ 0.5–8.5) were binned within each 0.002-ppm wide region and automatically integrated with the AMIX 3.8.3 package (Bruker Biospin Corp., Billerica, MA, USA). The region δ 4.54–5.20 was removed to avoid the effects of imperfect water suppression. Consequently, spectra over the δ 0.5–4.54 and 5.20–8.50 ranges were selected and reduced to 3,671 regions with 0.002-ppm width. Each integral region was normalized to the sum of all integral regions for each spectrum prior to pattern recognition analyses. An overview of data distribution and inter-sample similarities (i.e., clustering and outliers) for each sample was firstly investigated by principal component analysis (PCA), which was performed with the Simca-P 12.0 (Umetrics, Umeå, Sweden) software (13).

### Statistical Analysis

Data were analyzed by multifactorial analysis of variance (ANOVA) using the general linear model (GLM) procedure of SAS 9.1 software (SAS Institute Inc., Cary, NC, USA), followed by Tukey's tests to examine the significance of differences among means. The effects of physiological stages, breeds, dietary nutrient levels, and their interactions were all taken into account. Log transformation of variables was performed when the variance of data was not homogenous among treatment groups, which was assessed using Levene's test (14). Results are presented as means ± pooled standard error of the mean (SEM). Effects were considered statistically significant at *p* < 0.05, and *p-*values between 0.05 and 0.10 were considered trends.

## Results

### Contents of Amino Acids of *Psoas Major* Muscle

As shown in Table 3, the contents of each AA, total AAs (TAAs), essential AAs (EAAs), and flavor-related AAs (FAAs) of PMM were increased (*p* < 0.05) as age increased. Compared with the nursery stage, the content of non-essential AAs (NEAAs) was decreased (*p* < 0.05) in the growing stage in Bama mini-pigs while increased (*p* < 0.05) in the finishing stage in Landrace pigs and Bama mini-pigs. Ratios of EAA to TAA and of EAA to NEAA were increased in the growing stage but decreased in the finishing stage (*p* < 0.05). Compared with Landrace pigs, Bama mini-pigs showed higher contents of leucine (Leu), methionine (Met), phenylalanine (Phe), proline (Pro), serine (Ser), and tyrosine (Tyr) in both nursery and growing stages and lower contents of the above-mentioned AAs in the finishing stage (*p* < 0.05). Landrace pigs presented a higher (*p* < 0.05) content of glutamate (Glu) than Bama mini-pigs during the growing and finishing stages. The NRC diet led to an increased (*p* < 0.05) threonine (Thr) content in Landrace pigs and Bama mini-pigs throughout the trial. The NRC diet also increased (*p* < 0.05) the contents of histidine (His) and Ser in Bama mini-pigs throughout the three development stages, and in Landrace pigs in the nursery stage, when compared with the GB diet. Significant interactions (*p* < 0.05) were detected between physiological stage and breed for most AAs, such as alanine (Ala), aspartate (Asp), Glu, Thr, and Ser; and the diet effect interacted with that of breed regarding His content.

**Table 3:** Contents of amino acids in *psoas major* muscle of pigs.

**Items**	**Nursery stage**				**Growing stage**				**Finishing stage**				**SEM**	***p*** **-values**					
	**Landrace pig**		**Bama mini-pig**		**Landrace pig**		**Bama mini-pig**		**Landrace pig**		**Bama mini-pig**			***p*_**S**_**	***p*_**B**_**	***p*_**D**_**	***p*_**S × B**_**	***p*_**S × D**_**	***p*_**B × D**_**
	**GB diet**	**NRC diet**	**GB diet**	**NRC diet**	**GB diet**	**NRC diet**	**GB diet**	**NRC diet**	**GB diet**	**NRC diet**	**GB diet**	**NRC diet**							
Ala	13.08	14.23	13.22	13.97	10.94	9.72	14.48	14.75	24.73	30.24	18.55	20.76	1.87	<0.01	0.34	0.25	<0.01	0.39	0.77
Arg	10.62	10.66	10.85	11.42	12.42	12.01	12.28	12.51	13.11	13.22	12.02	13.05	0.28	<0.01	0.93	0.17	0.05	0.38	0.07
Asp	16.03	16.25	16.63	17.60	19.08	18.31	19.40	19.63	20.69	20.99	18.87	20.33	0.44	<0.01	0.54	0.18	<0.01	0.29	0.11
Glu	32.63	33.02	33.20	35.00	39.90	39.75	36.74	37.52	40.33	40.36	35.84	38.76	0.96	<0.01	0.02	0.14	<0.01	0.77	0.18
Gly	7.79	7.57	7.85	8.12	8.30	8.54	8.90	9.09	9.22	9.31	8.93	9.22	0.22	<0.01	0.12	0.32	0.12	0.84	0.47
His	6.79	6.91	6.52	7.14	8.43	8.07	7.96	9.09	8.73	9.34	8.41	9.79	0.31	<0.01	0.61	<0.01	0.84	0.40	0.03
Ile	6.79	6.26	7.18	7.61	8.77	8.55	8.27	7.77	8.68	8.84	7.92	8.31	0.26	<0.01	0.44	0.80	<0.01	0.37	0.39
Leu	14.32	14.15	16.39	17.16	16.72	16.00	19.83	18.08	19.01	19.02	17.67	18.47	0.70	<0.01	<0.01	0.70	<0.01	0.30	0.80
Lys	14.43	14.69	14.97	15.89	17.29	16.65	16.92	17.25	18.27	18.49	16.78	18.03	0.39	<0.01	0.99	0.14	0.02	0.36	0.10
Met	2.49	3.00	6.08	5.87	3.60	2.74	4.11	3.46	6.72	5.65	3.95	5.79	0.48	<0.01	0.01	0.83	<0.01	0.34	0.22
Phe	6.05	6.02	6.94	7.21	6.96	6.80	7.75	7.31	8.04	8.06	7.24	7.99	0.21	<0.01	<0.01	0.64	<0.01	0.16	0.38
Pro	8.93	8.06	40.64	38.51	10.23	9.11	20.46	10.62	31.92	30.48	21.19	25.49	3.53	<0.01	<0.01	0.44	<0.01	0.52	0.77
Ser	6.17	6.79	6.89	7.24	6.98	6.97	7.76	7.96	8.30	8.27	7.57	8.20	0.20	<0.01	<0.01	0.03	<0.01	0.46	0.44
Thr	8.20	8.55	8.62	9.07	9.91	9.97	9.74	10.05	10.64	10.80	9.61	10.51	0.23	<0.01	0.62	0.02	0.02	0.69	0.25
Tyr	5.07	5.55	6.51	6.82	4.77	4.26	7.20	5.43	6.98	6.64	5.56	6.77	0.45	0.02	<0.01	0.73	<0.01	0.06	0.95
Val	8.22	7.94	7.55	8.10	9.45	8.13	8.71	9.12	9.83	10.42	9.36	9.65	0.34	<0.01	0.29	0.86	0.45	0.31	0.11
TAA	167.60	169.64	210.06	216.73	193.75	185.58	210.51	199.64	245.19	250.11	209.45	231.10	6.97	<0.01	0.02	0.57	<0.01	0.16	0.51
EAA	77.91	78.17	85.11	89.47	93.53	88.92	95.57	94.63	103.03	103.83	92.95	101.58	2.25	<0.01	0.13	0.35	<0.01	0.14	0.09
NEAA	89.69	91.47	124.95	127.26	100.21	96.65	114.94	105.01	142.16	146.28	116.50	129.52	5.16	<0.01	0.02	0.71	<0.01	0.23	0.88
FAA	80.14	81.73	81.76	86.11	90.65	88.33	91.81	93.50	108.08	114.11	94.20	102.11	2.79	<0.01	0.23	0.09	<0.01	0.32	0.45
EAA/TAA	0.47	0.46	0.41	0.41	0.48	0.48	0.46	0.47	0.42	0.42	0.45	0.44	0.01	<0.01	<0.01	0.99	<0.01	0.57	0.32
EAA/NEAA	0.87	0.86	0.68	0.71	0.93	0.92	0.85	0.90	0.73	0.72	0.81	0.80	0.02	<0.01	<0.01	0.81	<0.01	0.72	0.33

*Values are expressed as mg/g fresh muscle tissue (means with pooled SEM), n = 8 per treatment group*.

*S, stage; B, breed; D, diet; S × B, stage × breed interaction; S × D, stage × diet interaction; B × D, breed × diet interaction*.

*Ala, alanine; Arg, arginine; Asp, asparate; Glu, glutamate; Gly, glycine; His, histidine; Ile, isoleucine; Lys, lysine; Met, methionine; Phe, phehylalanine; Pro, proline; Ser, serine; Thr, threonine; Tyr, tyrosine; Val, valine*.

*TAA, total amino acids; EAA, essential amino acids, including arginine, histidine, isoleucine, leucine, lysine, methionine, phenylalanine, threonine, and valine; NEAA, non-essential amino acids, including alanine, aspartate, glutamate, glycine, proline, serine, and tyrosine; FAA, flavor-related amino acids, including alanine, arginine, aspartate, glutamate, and glycine; EAA/TAA, ratio of EAA to TAA; EAA/NEAA, ratio of EAA to NEAA*.

### Composition of Fatty Acids of *Psoas Major* Muscle

As shown in Table 4, the percentages of C14:0, C18:1, and monounsaturated FAs (MUFAs) were increased gradually in PMM, while those of C18:3*n*−3, C20:4*n*−6, C20:5*n*−3, C22:6*n*−3, and polyunsaturated FAs (PUFAs) were decreased throughout the trial (*p* < 0.05). The percentages of C16:1, C18:2, C20:4*n*−6, C20:5*n*−3, C22:6*n*−3, and PUFA were higher, while those of C18:1, C18:3*n*−3, saturated FAs (SFAs), and MUFA were lower in Landrace pigs than in Bama mini-pigs (*p* < 0.05). The percentages of C18:0 in the nursery stage and C14:0 and C18:0 in the finishing stage were higher (*p* < 0.05) in Bama mini-pigs than in Landrace pigs. The NRC diet increased (*p* < 0.05) the percentages of C20:5*n*−3 and C22:6*n*−3 than did the GB diet. Significant interactions (*p* < 0.05) were found between physiological stage and diet for the percentages of C14:0, C16:0, C18:2, C20:4*n*−6, SFA, and PUFA, as well as between breed and diet for the percentage of C16:0.

**Table 4:** Percentages of fatty acids in *psoas major* muscle of pigs.

**Items**	**Nursery stage**				**Growing stage**				**Finishing stage**				**SEM**	***p*** **-values**					
	**Landrace pig**		**Bama mini-pig**		**Landrace pig**		**Bama mini-pig**		**Landrace pig**		**Bama mini-pig**			***p*_**S**_**	***p*_**B**_**	***p*_**D**_**	***p*_**S × B**_**	***p*_**S × D**_**	***p*_**B × D**_**
	**GB diet**	**NRC diet**	**GB diet**	**NRC diet**	**GB diet**	**NRC diet**	**GB diet**	**NRC diet**	**GB diet**	**NRC diet**	**GB diet**	**NRC diet**							
C14:0	0.99	1.15	0.96	1.18	1.22	1.14	1.20	1.30	1.24	1.20	1.39	1.34	0.05	<0.01	0.04	0.13	0.22	0.01	0.22
C16:0	28.37	29.34	26.68	30.45	28.70	26.05	27.50	27.92	27.21	25.81	27.94	27.70	0.93	0.08	0.45	0.81	0.54	0.03	0.05
C16:1	1.84	2.17	1.21	1.44	2.38	2.37	0.87	1.39	1.66	2.04	1.03	0.99	0.28	0.35	<0.01	0.20	0.43	0.97	1.00
C18:0	10.88	15.60	15.93	14.73	16.00	14.41	16.72	16.12	15.52	14.22	17.23	16.52	1.16	0.14	0.02	0.88	0.87	0.20	0.33
C18:1	30.61	31.51	33.91	40.28	31.43	34.30	43.16	41.71	36.80	37.67	43.30	41.81	1.74	<0.01	<0.01	0.23	0.27	0.32	0.86
C18:2	19.95	13.57	15.38	6.54	16.38	17.23	8.09	8.27	13.57	15.59	6.96	8.58	1.77	0.16	<0.01	0.13	0.59	<0.01	0.61
C18:3*n*−3	0.99	1.04	1.42	1.60	0.77	0.76	1.37	1.35	0.90	0.74	1.18	1.06	0.14	0.03	<0.01	0.89	0.44	0.55	0.78
C20:0	0.21	0.23	0.18	0.22	0.24	0.19	0.20	0.24	0.21	0.20	0.20	1.06	0.20	0.32	0.27	0.24	0.28	0.32	0.19
C20:4*n*−6	4.71	3.31	3.34	2.30	2.28	2.44	0.74	1.23	2.38	1.72	0.64	0.68	0.39	<0.01	<0.01	0.12	0.93	0.04	0.36
C20:5*n*−3	0.44	0.62	0.34	0.48	0.21	0.42	0.06	0.16	0.15	0.21	0.04	0.08	0.04	<0.01	<0.01	<0.01	0.37	0.17	0.25
C22:6*n*−3	1.02	1.47	0.66	0.78	0.40	0.71	0.10	0.31	0.38	0.59	0.13	0.19	0.08	<0.01	<0.01	<0.01	0.25	0.47	0.08
SFA	40.45	46.32	43.75	46.58	46.17	41.78	45.61	45.58	44.18	41.42	46.46	46.63	1.42	0.89	0.01	0.76	0.58	<0.01	0.44
MUFA	32.44	33.67	35.12	41.72	33.81	36.67	44.03	43.10	38.46	39.72	44.33	42.79	1.71	<0.01	<0.01	0.15	0.34	0.29	0.85
PUFA	27.11	20.01	21.14	11.70	20.02	21.55	10.36	11.32	17.37	18.86	8.96	10.58	1.92	<0.01	<0.01	0.14	0.64	<0.01	0.71

*Values are expressed as % (means with pooled SEM), n = 8 per treatment group*.

*S, stage; B, breed; D, diet; S × B, stage × breed interaction; S × D, stage × diet interaction; B × D, breed × diet interaction*.

*SFA, saturated fatty acids, including C14:0, C16:0, C18:0, and C20:0; MUFA, monounsaturated fatty acids, including C16:1 and C18:1; PUFA, polyunsaturated fatty acids, including C18:2, C18:3n−3, C20:4n−6, C20:5n−3, and C22:6n−3*.

### Multivariate Data Analysis of NMR Data

**Figure 1:**
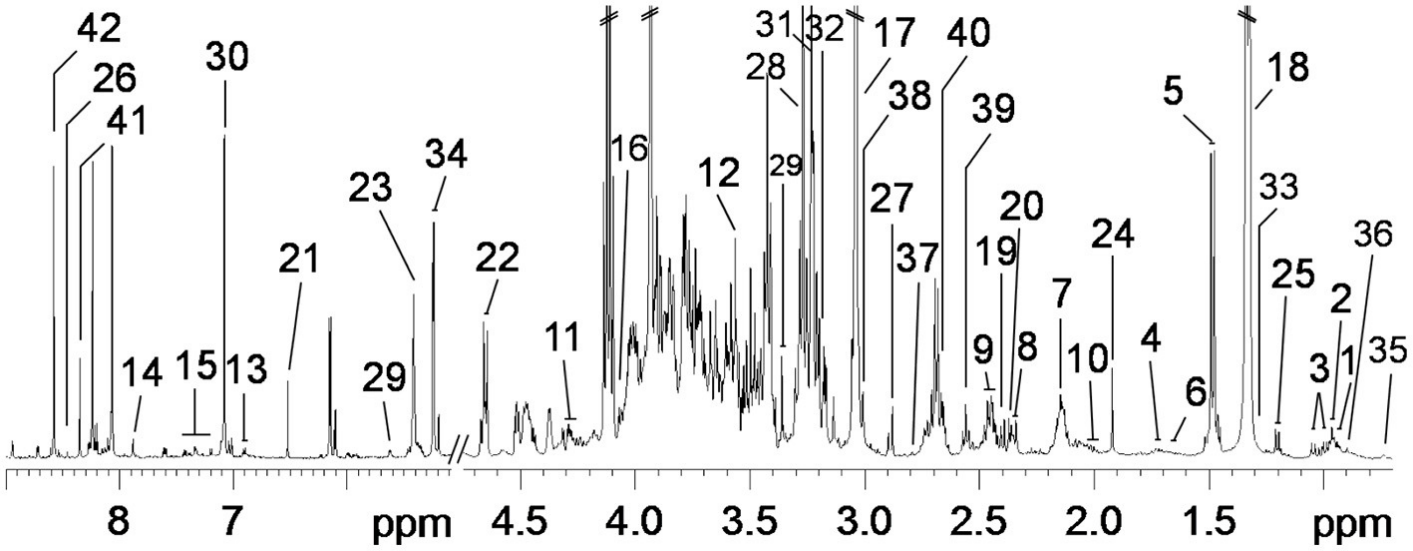
Typical 600-MHz ^1^H NMR spectra from *psoas major* muscle of pigs. A total of 42 metabolites were unambiguously assigned. 1, isoleucine; 2, leucine; 3, valine; 4, lysine; 5, alanine; 6, arginine; 7, methionine; 8, glutamate; 9, glutamine; 10, proline; 11, threonine; 12, glycine; 13, tyrosine; 14, 1-methylhistidine; 15, phenylalanine; 16, creatine; 17, creatinine; 18, lactate; 19, pyruvate; 20, succinate; 21, fumarate; 22, β-glucose; 23, α-glucose; 24, acetate; 25, β-hydroxyisobutyrate; 26, formate; 27, trimethylamine; 28, trimethylamine oxide (TMAO); 29, *myo*-Inositol; 30, histidine; 31, choline; 32, glycerol-phosphatidylcholine (GPC); 33, lipids (triglycerides and fatty acids); 34, unsaturated lipids; 35, low-density lipoprotein (LDL); 36, very low-density lipoprotein (VLDL); 37, dimethylamine; 38, albumin; 39, α-ketoglutarate; 40, aspartate; 41, guanine; 42, adenosine.

**Figure 2:**
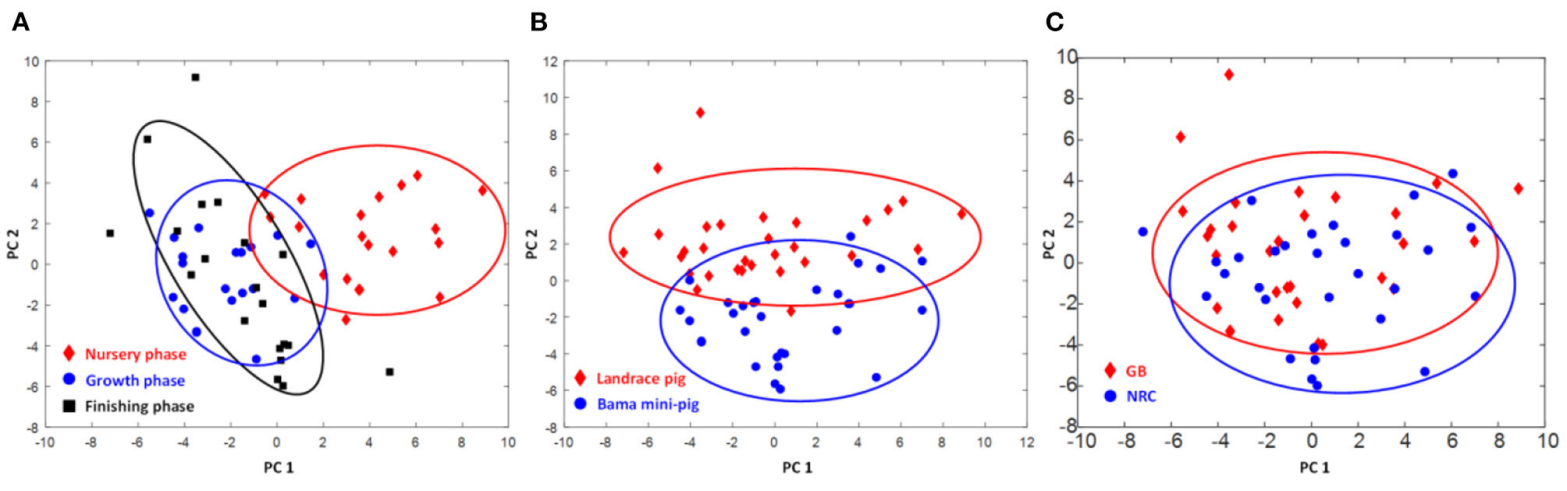
Score plots of metabolome of *psoas major* muscle of pigs. **(A)** Different physiological stages (PC1 = 0.266, PC2 = 0.253, *Q*^2^ = 0.368); **(B)** different breeds of pigs (PC1 = 0.253, PC2 = 0.519, *Q*^2^ = 0.368); **(C)** different diets (PC1 = 0.266, PC2 = 0.253, *Q*^2^ = 0.368).

The contents of glycerol-phosphatidylcholine (GPC) of PMM in Landrace pigs and His in Bama mini-pigs were increased (*p* < 0.05), while those of lysine (Lys), acetate, trimethylamine, trimethylamine oxide (TMAO), *myo*-inositol, glycine (Gly), Thr, fumarate, and Glu in Landrace pigs and α-glucose in Bama mini-pigs were decreased gradually throughout the trial (Figure 1 and Table 5). When compared with the nursery stage, the contents of Lys, acetate, and pyruvate were decreased in the growing stage but increased in the finishing stage in Bama mini-pigs (*p* < 0.05). In addition, the metabolic profiles in the nursery stage were significantly different from those in both growing and finishing stages (Figure 2).

**Table 5:** Metabolome of *psoas major* muscle of pigs.

**Items**	**Nursery stage**				**Growing stage**				**Finishing stage**				**SEM**	***p*** **-values**					
	**Landrace pig**		**Bama mini-pig**		**Landrace pig**		**Bama mini-pig**		**Landrace pig**		**Bama mini-pig**			***p*_**S**_**	***p*_**B**_**	***p*_**D**_**	***p*_**S × B**_**	***p*_**S × D**_**	***p*_**B × D**_**
	**GB diet**	**NRC diet**	**GB diet**	**NRC diet**	**GB diet**	**NRC diet**	**GB diet**	**NRC diet**	**GB diet**	**NRC diet**	**GB diet**	**NRC diet**							
LDL (0.84)	0.10	0.15	0.12	0.14	0.09	0.13	0.12	0.10	0.08	0.09	0.14	0.17	0.01	0.24	<0.01	0.01	<0.01	0.46	0.19
VLDL (0.88)	0.38	0.55	0.38	0.43	0.32	0.42	0.42	0.39	0.31	0.33	0.45	0.51	0.05	0.48	0.16	0.06	0.03	0.58	0.25
Leu (0.96)	0.53	0.54	0.63	0.69	0.44	0.56	0.63	0.59	0.37	0.51	0.62	0.68	0.04	0.14	<0.01	0.01	0.13	0.35	0.16
Ile (1.01)	0.22	0.24	0.30	0.32	0.19	0.24	0.29	0.27	0.17	0.24	0.29	0.32	0.02	0.26	<0.01	<0.01	0.36	0.38	0.08
Val (1.04)	0.29	0.30	0.39	0.44	0.25	0.32	0.40	0.36	0.24	0.33	0.40	0.41	0.02	0.43	<0.01	0.01	0.62	0.52	0.04
β-Hydroxy-isobutyrate (1.20)	0.62	0.76	0.66	0.71	0.63	0.65	0.67	0.69	0.71	0.70	0.66	0.64	0.04	0.53	0.77	0.13	0.25	0.08	0.39
Lipids (1.29)	0.90	0.96	0.98	1.00	0.79	0.89	0.99	1.05	1.02	1.07	0.95	1.03	0.08	0.30	0.21	0.17	0.11	0.93	0.89
Ala (1.48)	3.77	3.62	4.17	4.33	2.24	3.14	1.92	2.38	2.32	2.61	2.49	3.43	0.31	<0.01	0.34	0.02	0.03	0.24	0.64
Arg (1.63)	0.44	0.48	0.51	0.54	0.40	0.52	0.52	0.46	0.32	0.37	0.54	0.62	0.03	0.31	<0.01	0.01	<0.01	0.63	0.12
Lys (1.73)	0.72	0.82	0.84	0.92	0.60	0.78	0.78	0.76	0.58	0.66	0.83	0.98	0.04	0.01	<0.01	<0.01	<0.01	0.85	0.31
Acetate (1.92)	0.64	0.64	0.68	0.70	0.48	0.58	0.48	0.44	0.43	0.36	0.59	1.02	0.07	<0.01	<0.01	0.07	<0.01	0.17	0.11
Pro (2.02)	0.40	0.48	0.44	0.48	0.32	0.39	0.36	0.34	0.27	0.29	0.41	0.46	0.03	<0.01	<0.01	0.02	<0.01	0.64	0.23
Met (2.13)	0.94	1.29	1.23	1.28	0.80	1.07	1.06	1.04	0.96	0.77	1.48	1.36	0.09	0.01	<0.01	0.31	<0.01	0.02	0.11
Glu (2.35)	1.17	1.26	1.34	1.40	0.73	0.79	0.75	0.85	0.75	0.71	0.84	1.05	0.09	<0.01	0.02	0.15	0.40	0.99	0.40
Pyruvate (2.37)	0.30	0.30	0.33	0.33	0.19	0.23	0.19	0.24	0.25	0.23	0.25	0.30	0.02	<0.01	0.12	0.19	0.59	0.37	0.35
Succinate (2.40)	0.36	0.42	0.67	0.42	0.26	0.35	0.30	0.41	0.56	0.38	0.42	0.47	0.09	0.06	0.25	0.71	0.39	0.27	0.83
Glu (2.45)	1.91	2.11	2.49	2.45	1.36	1.92	1.70	1.74	1.48	1.36	2.72	2.38	0.22	<0.01	<0.01	0.70	<0.01	0.25	0.21
α-Ketoglutarate	0.92	1.03	0.98	0.89	0.82	0.86	0.73	0.79	0.74	0.83	0.84	1.04	0.06	<0.01	0.72	0.04	0.01	0.24	0.79
Asp (2.7)	7.83	6.90	8.28	9.00	10.27	8.90	10.30	11.14	9.40	10.76	10.28	11.21	0.49	<0.01	<0.01	0.37	0.66	0.10	0.05
Dimethylamine (2.72)	0.25	0.24	0.31	0.28	0.33	0.31	0.37	0.27	0.15	0.21	0.31	0.34	0.02	<0.01	<0.01	0.36	<0.01	0.01	0.14
Trimethylamine (2.88)	0.61	0.39	0.49	0.55	0.41	0.42	0.39	0.34	0.29	0.29	0.38	0.36	0.07	<0.01	0.68	0.37	0.44	0.75	0.44
Albumin (3.02)	1.20	1.11	1.39	1.37	1.55	1.47	1.70	1.64	1.38	1.63	1.54	1.71	0.06	<0.01	<0.01	0.43	0.47	<0.01	0.90
Creatinine (3.05)	2.26	2.17	2.38	2.25	2.82	2.67	2.98	2.91	2.75	2.80	2.71	2.93	0.11	<0.01	0.09	0.65	0.60	0.23	0.59
Choline (3.20)	3.93	3.56	4.16	4.38	5.41	4.68	5.64	5.66	4.41	5.06	5.64	6.35	0.25	<0.01	<0.01	0.57	0.09	0.02	0.12
GPC (3.22)	5.13	4.69	5.23	5.60	6.01	5.61	6.35	6.69	5.53	6.26	6.80	6.79	0.24	<0.01	<0.01	0.49	0.51	0.42	0.34
TMAO (3.26)	5.86	5.39	5.11	3.88	4.68	4.21	2.91	2.80	4.21	3.59	3.46	2.80	0.37	<0.01	<0.01	<0.01	0.31	0.56	0.74
*myo*-Inositol (3.35)	1.51	1.36	0.83	1.00	0.80	0.86	0.85	0.45	0.30	0.37	0.84	0.77	0.18	<0.01	0.46	0.62	<0.01	0.74	0.64
Gly (3.56)	2.34	2.38	2.28	2.37	1.27	1.64	1.34	1.38	1.21	1.40	1.33	1.39	0.14	<0.01	0.75	0.11	0.75	0.77	0.39
Creatine (δ 3.93)	24.61	25.83	22.60	22.31	23.69	24.35	22.95	22.28	30.49	24.69	21.46	21.33	1.51	0.55	<0.01	0.34	0.08	0.23	0.59
Lactate (4.11)	17.62	17.86	17.78	17.83	20.28	18.91	20.10	19.59	17.85	19.38	19.07	16.23	0.63	<0.01	0.55	0.19	0.35	0.45	0.10
Thr (4.30)	0.97	0.94	0.84	0.86	0.65	0.76	0.81	0.67	0.52	0.73	0.64	0.67	0.05	<0.01	0.64	0.29	0.07	0.11	0.03
α-Glucose (5.23)	2.68	2.87	2.79	2.12	1.16	2.05	1.30	1.27	1.24	1.37	0.76	0.36	0.15	<0.01	<0.01	0.82	0.08	<0.01	<0.01
β-Glucose (6.10)	0.33	0.29	0.33	0.29	0.20	0.28	0.20	0.18	0.21	0.23	0.27	0.23	0.02	<0.01	0.75	0.65	0.06	0.08	0.07
Unsaturated lipids (6.13)	1.64	1.55	1.35	1.42	1.63	1.70	1.70	1.66	1.52	1.60	1.60	1.36	0.08	<0.01	0.04	0.50	0.12	0.67	0.33
Fumarate (6.52)	0.18	0.14	0.16	0.14	0.09	0.10	0.05	0.08	0.08	0.08	0.08	0.10	0.02	<0.01	0.32	0.94	0.31	0.13	0.42
Tyr (6.88)	0.12	0.13	0.13	0.15	0.11	0.12	0.12	0.13	0.08	0.11	0.12	0.13	0.01	<0.01	<0.01	<0.01	0.03	0.59	0.52
His (7.08)	3.91	3.33	4.21	4.51	5.49	4.67	5.20	5.49	4.68	5.33	5.11	5.17	0.25	<0.01	0.01	0.91	0.21	0.19	0.12
Phe (7.42)	0.14	0.17	0.13	0.15	0.11	0.14	0.12	0.13	0.12	0.12	0.12	0.15	0.01	<0.01	0.72	<0.01	0.25	0.89	0.85
1-Methylhistidine (7.76)	0.18	0.23	0.26	0.28	0.18	0.24	0.32	0.34	0.15	0.20	0.36	0.43	0.03	0.05	<0.01	0.01	<0.01	0.77	0.63
Guanine (8.35)	0.30	0.25	0.30	0.26	0.17	0.24	0.17	0.16	0.17	0.20	0.24	0.20	0.02	<0.01	0.96	0.59	0.05	0.05	0.09
Formate (8.45)	0.03	0.05	0.07	0.05	0.02	0.03	0.02	0.06	0.05	0.05	0.02	0.04	0.01	0.27	0.51	0.23	0.23	0.63	0.76
Adenosine (8.58)	1.52	1.41	1.24	1.34	1.52	1.58	1.59	1.56	1.41	1.49	1.52	1.29	0.07	<0.01	0.11	0.56	0.13	0.65	0.39

*Values are expressed as % (means with pooled SEM), n = 8 per treatment group*.

*S, stage; B, breed; D, diet; S × B, stage × breed interaction; S × D, stage × diet interaction; B × D, breed × diet interaction*.

*Ala, alanine; Arg, arginine; Asp, asparate; Glu, glutamate; Gly, glycine; GPC, glycerol-phospatidycholine; His, histidine; Ile, isoleucine; Leu, leucine; LDL, low-density lipoprotein; Lys, lysine; Met, methionine; Phe, phehylalanine; Pro, proline; Thr, threonine; TMAO, trimethylamine oxide; Tyr, tyrosine; Val, valine; VLDL, very low-density lipoprotein*.

*LDL, low-density lipoprotein; VLDL, very low-density lipoprotein; GPC, glycerol-phosphatidylcholine; TMAO, trimethylamine oxide*.

The contents of Leu, isoleucine (Ile), valine (Val), arginine (Arg), Lys, glutamine (Gln), dimethylamine, albumin, choline, GPC, and 1-methylhistidine were higher (*p* < 0.05) in Bama mini-pigs, while those of TMAO and creatine were lower (*p* < 0.05) than in Landrace pigs throughout the trial. In the finishing stage, the contents of Met and Pro were higher and those of α-glucose were lower in Bama mini-pigs than in Landrace pigs (*p* < 0.05).

When compared with the GB diet, the NRC diet increased the contents of low-density lipoprotein (LDL), Ala, and 1-methylhistidine and decreased those of TMAO in the PMM of the two pig breeds (*p* < 0.05). The NRC diet also increased (*p* < 0.05) the contents of α-ketoglutarate throughout the three stages; Leu, Arg, and α-glucose in the growing stage of Landrace pigs; α-ketoglutarate in the growing stage; and acetate and α-ketoglutarate in the finishing stage of Bama mini-pigs, but decreased (*p* < 0.05) the contents of choline in the growing stage and creatine in the finishing stage of Landrace pigs, as well as Leu and Arg in the growing stage of Bama mini-pigs.

## Discussion

Animals have dietary requirements regarding not only EAA but also NEAA to achieve maximum growth and production performance (15, 16). Skeletal muscle represents 40–45% of the body's weight and is the largest reservoir of both peptide-bound and free AAs in the body (17). In the present study, the contents of most AAs, TAA, EAA, and FAA in the PMM increased in an age-dependent manner, regardless of pig breeds and dietary nutrient levels, indicating an enhanced deposition of muscular protein as pigs matured. In addition, regardless of the GB or NRC diet, Bama mini-pigs showed higher contents of Leu, Met, Phe, Ser, and Tyr in both the nursery and growing stages, but lower contents of the above-mentioned AAs in the finishing stage than in the Landrace pigs. Indeed, Leu, Met, Phe, Ser, and Tyr are indispensable AAs or precursor substances for palatable meats (18). Our findings suggest that the muscle of Bama mini-pigs in both nursery and growing stages can generate more precursor substances, which is preferable for the production of delicious meat, in terms of organoleptic characteristics. Furthermore, compared with the GB diet, the NRC diet increased the muscular His and Thr contents in both pig breeds throughout the trial. Moreover, the NRC diet increased the Ser content in Bama mini-pigs throughout the three stages and the nursery stage in Landrace pigs, indicating that a high level of dietary protein increased protein deposition in the skeletal muscle of pigs (19).

There is a negative correlation between PUFA and meat flavor or overall acceptability, and a positive correlation between MUFA and meat flavor or overall acceptability (20). Moreover, the PUFA content is related to the nutritional value of meat (21, 22). In the present study, the contents of C14:0, C18:1, and MUFA in the PMM of both pig breeds were increased, while those of C18:3*n*−3, C20:4*n*−6, C20:5*n*−3, C22:6*n*−3, and PUFA were decreased with increasing age, indicating better meat quality as the pigs matured. The FAs in the pig's carcass originate from two sources: whereas some, mostly SFA and MUFA, are synthesized by the pig, others, especially PUFA, are obtained from the diet and directly deposited in the tissues (23). In the present study, the percentages of SFA and MUFA were higher in Bama mini-pigs than in the Landrace pigs, indicating that the first exhibit higher synthesis ability for SFA and MUFA. The percentage of linoleic acid (C18:2) in the muscle declines as fat deposition proceeds, and therefore, it is considered an index of fatness (24). In the present study, Bama mini-pigs contained a lower content of C18:2 than in the Landrace pigs, indicating that Bama mini-pigs tend to have higher fat deposition in muscle. The NRC diet increased the contents of C20:5*n*−3 and C22:6*n*−3 in the PMM compared with the GB diet, indicating that a high level of dietary protein enhances swine's ability to synthesize PUFA.

Metabolomics may help to elucidate mechanisms underlying diet–disease relationships and identify novel risk factors for disease. To inform the design and interpretation of such research, evidence on diet–metabolite associations and cross-assay comparisons is needed (25). NMR method has the advantages of good reproducibility, good qualitative and quantitative analysis, low detection cost, and so on (26). In this study, we chose NMR-based metabolomics analysis technology. For analysis of the data extracted from NMR spectra, in general, principal component analysis (PCA), partial least squares (PLS), or multiple linear regression (MLR) are commonly employed. PCA is the most frequently employed exploratory data analysis technique, since it enables the identification of patterns and visualization of data distribution (27). Aging is a very complex process in animals because many biochemical processes occur from cells to organs, leading to a wide variety of altered biochemical functions (28, 29). This requires novel approaches for the phenotypic characterization of the gradual development of age-related chronic disorders at epidemiological and individual scales. Metabolomics, the quantitative measurement of the dynamic multiparametric metabolic response of living systems to pathophysiological stimuli or genetic modifications, provides a systematic approach for understanding organisms' global metabolic regulations (30, 31). In the present study, pigs' growth stage and breed significantly affected the metabolic profile of the skeletal muscle, while diet had only a significant impact on a few metabolites in the muscular tissue. Interestingly, regardless of pig breed, the metabolic profiles in the nursery stage were significantly different from those in both growing and finishing stages, suggesting that the circulation, distribution, and deposition of nutrients in muscle tend to attain a steady state in the growing and finishing stages. Furthermore, the contents of nitrogen-related products (i.e., albumin, Ala, Arg, Lys, Pro, Gln, α-ketoglutaric acid, and Gly) and intermediate metabolites of the methylamine pathway (i.e., dimethylamine and TMAO) were increased with growth, suggesting that most of these products may migrate into the skeletal muscle where they promote protein synthesis and deposition (32).

This study has also shown that the two breeds of pigs have distinct muscular metabolic profiles. The PMM contents of AA-related products, hydrolyzed AAs, and 1-methylhistidine were higher in Bama mini-pigs than in the Landrace pigs throughout the trial. Compared with Bama mini-pigs, Landrace pigs had more intermediate metabolites of the methylamine pathway, such as trimethylamine, TMAO, and creatinine, suggesting that the catabolism of lipids and cholesterol were increased in the skeletal muscle (33, 34). Previous studies showed that changes in the creatine metabolism in the skeletal muscle affect muscular glycol-metabolism (35), muscle fiber types, and meat quality (36). In the present study, Landrace pigs presented a higher muscular creatine content than in the Bama mini-pigs, which is closely related to their ability to synthesize protein and high percentage of lean meat.

Dietary nutrient levels have significant effects on the metabolites of animals (37, 38). In the present study, the NRC diet increased the α-ketoglutarate content in the Landrace pigs throughout the trial and in the Bama mini-pigs in the growing and finishing stages. The metabolite α-ketoglutarate is synthesized from glucose or oxaloacetate plus pyruvate (39) and plays important roles in cell metabolism and physiology. As an intermediate of the tricarboxylic acid cycle, α-ketoglutarate is essential for the oxidation of FAs, AAs, and glucose (40). It is also involved in the transamination of a variety of AAs, providing the carbon skeleton for the synthesis of Glu, Gln, Arg, and ornithine (41). Emerging evidence shows that α-ketoglutarate is a regulator of gene expression and cell signaling pathways (including the mammalian target of rapamycin and adenosine monophosphate-activated protein kinase) (41). Based on these findings, we hypothesize that a diet with high protein levels might increase the deposition of α-ketoglutarate in skeletal muscle, affecting muscle energy metabolism *via* certain signaling pathways, thereby improving body energy status.

In conclusion, the muscular contents of AAs and the percentages of MUFA in both pig breeds increased continuously, while the percentages of PUFA decreased with increasing age. Bama mini-pigs exhibited a higher ability to synthesize FAs and deposit fat than in the Landrace pigs. The NRC diet increased protein deposition in muscles but decreased meat flavor. These findings may have practical significance in how to use strategies to improve the nutritional value and flavor of the meat from pigs.

## Data Availability Statement

The original contributions presented in the study are included in the article/supplementary material, further inquiries can be directed to the corresponding author.

## Ethics Statement

The animal study was reviewed and approved by Animal Care and Use Committee of the Institute of Subtropical Agriculture, Chinese Academy of Sciences.

## Author Contributions

YL and QH conducted the animal trial, analyzed and interpreted the data, and wrote the paper. XK and YY conceived and designed the study. MA and YX assisted with tissue collection and data analysis. All authors read and approved the final manuscript.

## Conflict of Interest

The authors declare that the research was conducted in the absence of any commercial or financial relationships that could be construed as a potential conflict of interest.

## Publisher's Note

All claims expressed in this article are solely those of the authors and do not necessarily represent those of their affiliated organizations, or those of the publisher, the editors and the reviewers. Any product that may be evaluated in this article, or claim that may be made by its manufacturer, is not guaranteed or endorsed by the publisher.

## Abbreviations

AAs: amino acid
BW: body weight
EAA: essential amino acid
FAA: flavor amino acid
FA: fatty acid
GPC: glycerol-phosphatidylcholine
MUFA: monounsaturated fatty acid
NEAA: non-essential amino acid
NMR: nuclear magnetic resonance
PMM: *psoas major* muscle
PUFA: polyunsaturated fatty acid
SFA: saturated fatty acid
TAA: total amino acid
TMAO: trimethylamine oxide.
